# A Closed Glottis After Shoulder Arthroscopy: A Rare Airway Complication of Interscalene Block Causing Postoperative Respiratory Failure

**DOI:** 10.7759/cureus.111797

**Published:** 2026-06-30

**Authors:** Gonzalo J Martinez-Ruiz, Nickolle A Cruz-Figueroa, Luis Garcia-Requena, Bak N Choi-Reina, Jose A Torres-Cintron

**Affiliations:** 1 Internal Medicine, Central Caribbean University, Bayamon, USA

**Keywords:** acute hypoxemic respiratory failure, arthroscopic shoulder surgery, interscalene brachial plexus block, upper airway blockage, vocal cords

## Abstract

Interscalene brachial plexus block (ISB) is commonly used for shoulder surgery and is associated with predictable complications such as phrenic nerve palsy due to its proximity to adjacent neural structures. While the spread of local anesthetic may involve the recurrent laryngeal nerve, resulting in vocal cord dysfunction, which is uncommon, progression to bilateral vocal cord paralysis, causing acute airway obstruction, is exceedingly rare.

A 74-year-old obese female underwent elective outpatient right shoulder arthroscopy under ultrasound-guided interscalene block with bupivacaine. Within hours postoperatively, she developed progressive respiratory distress. Despite high-flow oxygen and bag-valve-mask ventilation, oxygen saturation declined into the 60s with marked resistance to ventilation. Emergent intubation resulted in immediate improvement. Point-of-care ultrasound demonstrated diffuse B-lines without right heart strain, and imaging confirmed pulmonary edema. Workup did not reveal a primary pulmonary or obstructive cardiac etiology; however, troponin elevation consistent with type 2 myocardial infarction was identified. The patient improved rapidly with ventilatory support and diuresis, was extubated within 48 hours, and was discharged without recurrence.

This case demonstrates a rare complication of ISB involving sequential spread of local anesthetic, potentially facilitated by arthroscopic fluid extravasation, leading to phrenic nerve involvement and bilateral recurrent laryngeal nerve dysfunction. Resultant airway obstruction caused negative pressure pulmonary edema and secondary demand ischemia.

Resistance to ventilation and refractory hypoxemia after ISB should raise concern for upper airway obstruction. Early airway management is critical to improve outcomes.

## Introduction

Interscalene brachial plexus block (ISB) is a regional anesthesia technique targeting the C5-C6 nerve roots between the anterior and middle scalene muscles and is widely used for shoulder surgery. It provides excellent analgesia, reduces opioid requirements, and facilitates faster recovery [1,2].

However, due to its anatomical location, ISB is associated with predictable complications. Phrenic nerve blockade occurs in up to 90% of cases with conventional anesthetic volumes, resulting in ipsilateral hemidiaphragmatic paresis [1,3].Although most patients tolerate this transient physiologic effect, cases of clinically significant respiratory compromise and severe hypoxemia requiring intensive care management have been reported[4]. Uncommonly, recurrent laryngeal nerve involvement may also occur, typically leading to hoarseness or unilateral vocal cord dysfunction [5,6].

Bilateral vocal cord paralysis resulting in acute airway obstruction is exceedingly rare, making recognition critical due to its life-threatening potential [7-9].

## Case presentation

A 74-year-old obese female with a history of type 2 diabetes mellitus, hypertension, hyperlipidemia, anxiety, and osteoarthritis underwent elective outpatient right shoulder arthroscopy for symptomatic osteoarthritis. Preoperative evaluation classified her as a low-risk patient undergoing a low-risk procedure, with well-controlled chronic medical conditions and no prior history of perioperative complications, including previous cholecystectomy and hysterectomy.

Preoperative laboratory studies, electrocardiogram, and chest radiograph were within normal limits (Table 1). She demonstrated functional capacity >4 metabolic equivalents of tasks (METs), with an Assess Respiratory Risk in Surgical Patients in Catalonia (ARISCAT) score of 3, revised cardiac risk index (RCRI) of 0, and Gupta perioperative cardiac risk of 0.3%, consistent with low perioperative risk. Snoring, tiredness, observed apnea, high blood pressure, body mass index, age, neck circumference, and gender (STOP-BANG) screening indicated low risk for obstructive sleep apnea [10-13].

**Table 1:** Preoperative Laboratory Evaluation Preoperative laboratory evaluation was unremarkable, demonstrating preserved hematologic, metabolic, and renal function without evidence of active infection, anemia, electrolyte disturbance, or end-organ dysfunction. Overall findings were consistent with a medically optimized patient undergoing elective surgery. BUN: blood urea nitrogen; eGFR: estimated glomerular filtration rate.

Preoperative lab test	Result	Reference Value	Units
White Blood Cells	8.6	4.8 – 11.0	x 10^3 /uL
Red Blood Cells	4.37	3.83 – 4.98	x 10^6 /uL
Hemoglobin	13.3	11.0 – 15.1	g/dL
Hematocrit	40.1	34.5 – 43.8	%
Platelets	269	161 - 400	x 10^3 /uL
Glucose	100	74 - 106	mg/dL
BUN	14.0	7.0 - 18	mg/dL
Creatinine	0.98	0.53 – 1.02	mg/dL
Sodium (Na)	138	136 - 145	mmol/L
Potassium (K)	5.1	3.5 – 5.1	mmol/L
Chloride (Cl)	102	98 - 107	mmol/L
Phosphorus	4.1	2.5 – 4.9	mg/dL
Carbon Dioxide (CO2)	22.9	21.0 – 32.0	mmol/L
Serum osmolality	280.68	275.0 – 295.0	mOsm/kgH2O
eGFR	> 60	> 60	mL/min/1.73m2

On the day of surgery, an ultrasound-guided interscalene brachial plexus block using bupivacaine was performed by the anesthesia team. The surgical procedure was completed without intraoperative complications.

In the immediate postoperative period, the patient developed mild to moderate respiratory distress with hypoxemia [87-90% peripheral oxygen saturation (SpO2)]. Internal medicine was consulted due to persistent dyspnea and lack of improvement with non-rebreather oxygen therapy, and she was admitted to the telemetry unit for further monitoring.

Upon arrival at the telemetry unit, the patient was in significant respiratory distress. She was nonverbal and able to communicate only through head nodding and arm gestures for yes/no responses. Within minutes, her clinical condition rapidly deteriorated, with increasing work of breathing and worsening hypoxemia. At that time, oxygen saturation acutely declined into the 60s despite high-flow oxygen and bag-valve-mask ventilation. Marked resistance to manual ventilation was noted, raising concern for imminent respiratory failure, and emergent endotracheal intubation was performed.

During intubation, significant resistance was encountered while advancing the endotracheal tube, and the vocal cords were noted to be tightly adducted in the midline without evidence of edema, consistent with upper airway obstruction. Following endotracheal intubation, oxygen saturation improved rapidly to 100%, and mechanical ventilation was achieved without further resistance. The patient was subsequently transferred to the intensive care unit for further management and evaluation of acute respiratory failure.

Investigations

Cardiopulmonary evaluation revealed no acute ischemic changes on electrocardiography. High-sensitivity troponin levels increased from 20.5 ng/L to a peak of 3,157.6 ng/L before subsequently declining to 2,405.5 ng/L. B-type natriuretic peptide (BNP) was elevated at 472 pg/mL, while D-dimer testing was negative (Table 2).

**Table 2:** Post-Admission Laboratory Evaluation Post-admission laboratory studies demonstrated mild leukocytosis and hyperglycemia, likely representing a physiologic stress response to acute illness. Renal function, serum osmolality, and electrolyte levels remained largely within normal limits. Cardiac biomarkers, including high-sensitivity troponin and creatine kinase-MB (CK-MB), were markedly elevated, consistent with myocardial injury. BNP was elevated, suggesting acute cardiac strain. D-dimer levels were within normal limits. BUN: blood urea nitrogen; eGFR: estimated glomerular filtration rate; CPK-MB: creatine phosphokinase-muscle/brain; BNP:  B-type natriuretic peptide.

Post-Admission lab test	Result	Reference Value	Units
White Blood Cells	13.2 - High	4.8 – 11.0	x 10^3 /uL
Red Blood Cells	4.03	3.83 – 4.98	x 10^6 /uL
Hemoglobin	12.6	11.0 – 15.1	g/dL
Hematocrit	37.0	34.5 – 43.8	%
Platelets	299	161 - 400	x 10^3 /uL
Glucose	195 - High	74 - 106	mg/dL
BUN	14.0	7.0 - 18	mg/dL
Creatinine	0.90	0.53 – 1.02	mg/dL
Sodium (Na)	137	136 - 145	mmol/L
Potassium (K)	5.0	3.5 – 5.1	mmol/L
Chloride (Cl)	105	98 - 107	mmol/L
Phosphorus	3.4	2.5 – 4.9	mg/dL
Carbon Dioxide (CO2)	19.1 - Low	21.0 – 32.0	mmol/L
Serum osmolality	279.65	275.0 – 295.0	mOsm/kgH2O
eGFR	> 60	> 60	mL/min/1.73m2
HS Troponin #1, 0 hours	20.5	0.0-58.9	pg/mL
HS Troponin #2, 8 hours	3157.6 – High	0.0-58.9	pg/mL
HS Troponin #3, 16 hours	2405.5 - High	0.0-58.9	pg/mL
CPK-MB	13.60 - High	0.50 – 3.60	ng/mL
D-Dimer	0.29	0.19 – 0.49	mg/L
BNP	472 - High	0.0 – 100.0	pg/mL

Additional laboratory studies, including a complete blood count and comprehensive metabolic panel, were within normal limits, with no evidence of infection, anemia, acute kidney injury, or significant electrolyte abnormalities.

**Figure 1:**
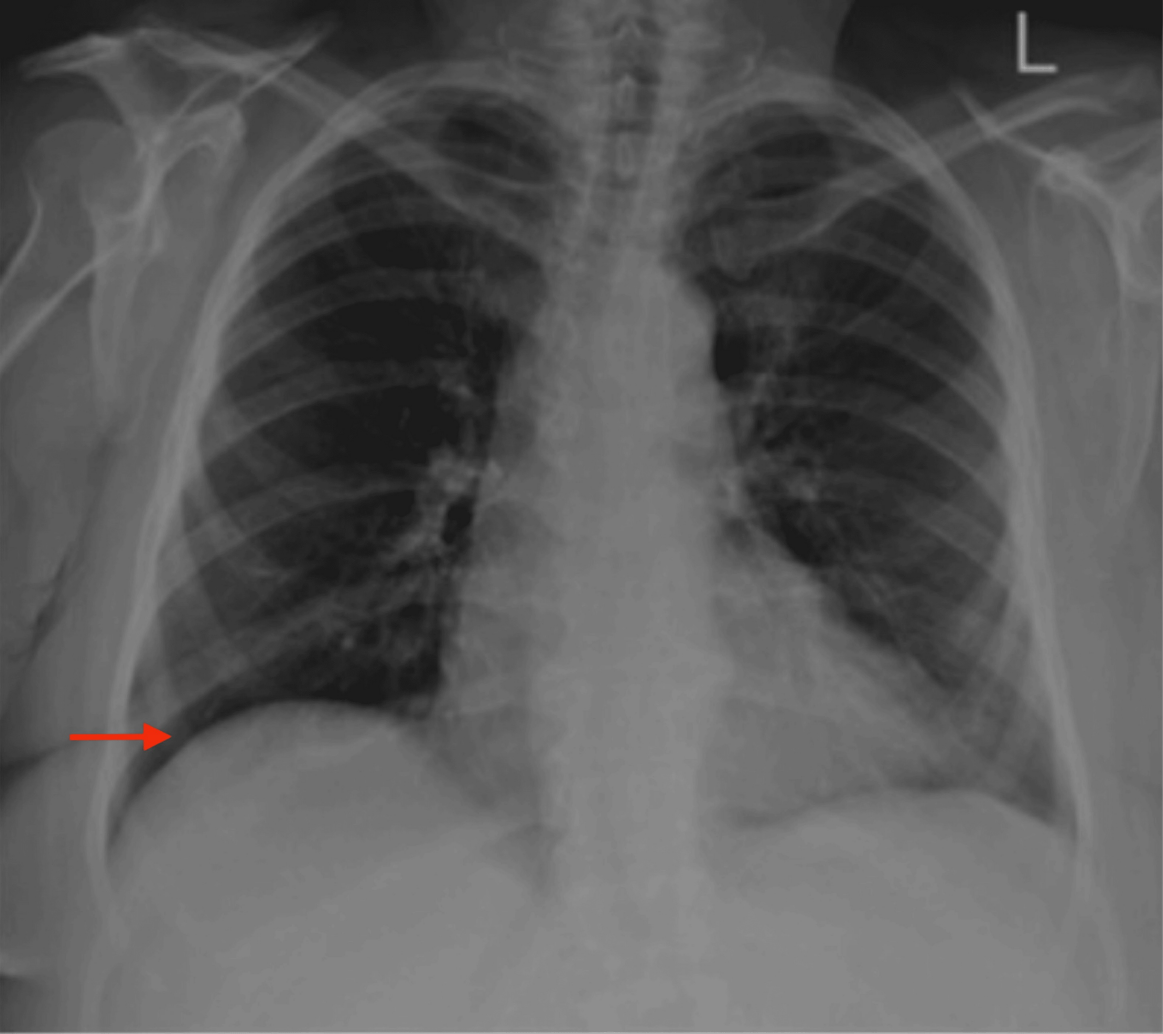
Preoperative Chest Radiograph Preoperative chest radiograph demonstrating no acute cardiopulmonary abnormality. The red arrow highlights a normally positioned right hemidiaphragm.

**Figure 2:**
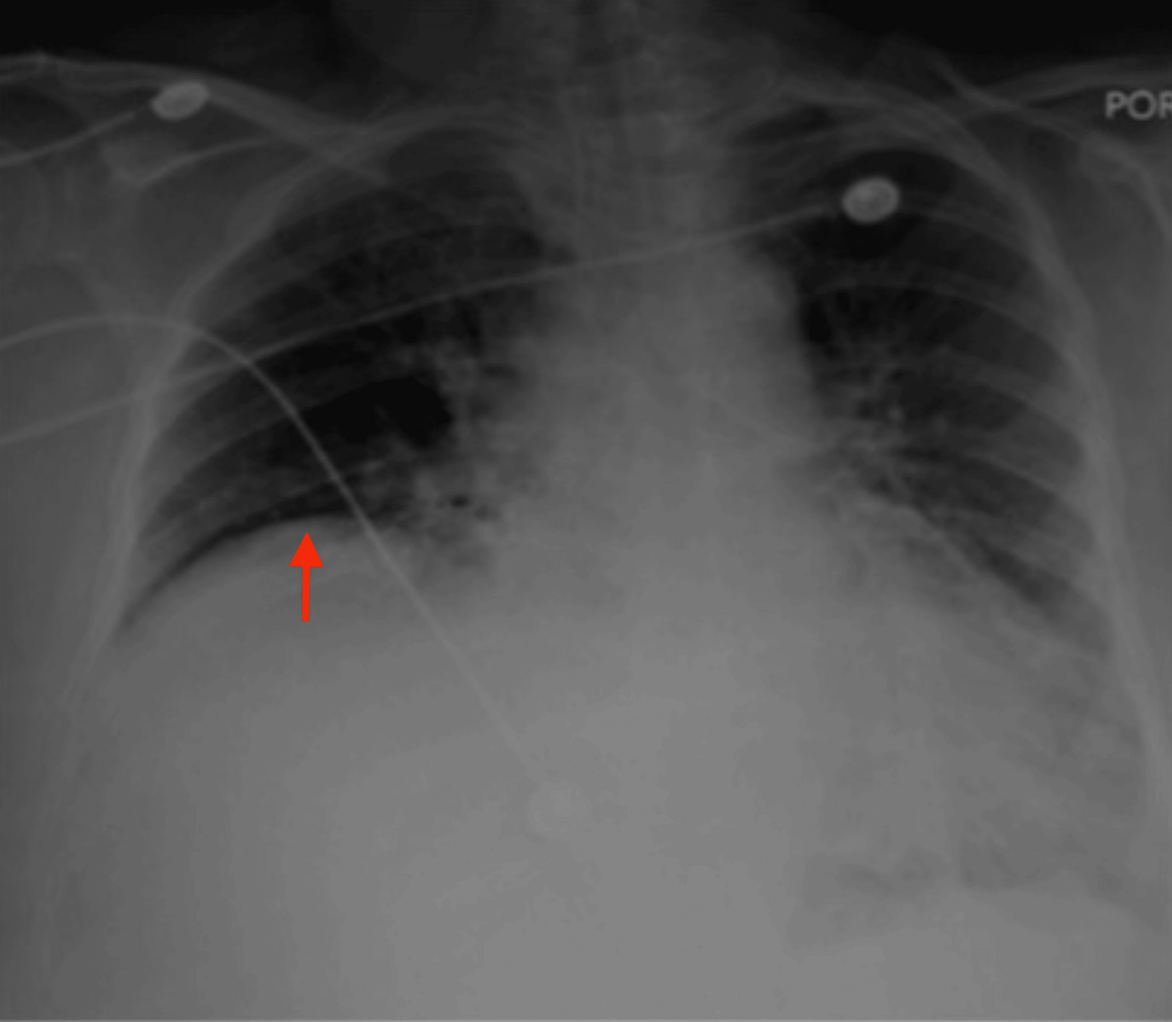
Postoperative Chest Radiograph Postoperative chest radiograph demonstrating new elevation of the right hemidiaphragm, suggestive of ipsilateral phrenic nerve dysfunction. The red arrow highlights the elevated right hemidiaphragm.

**Figure 3:**
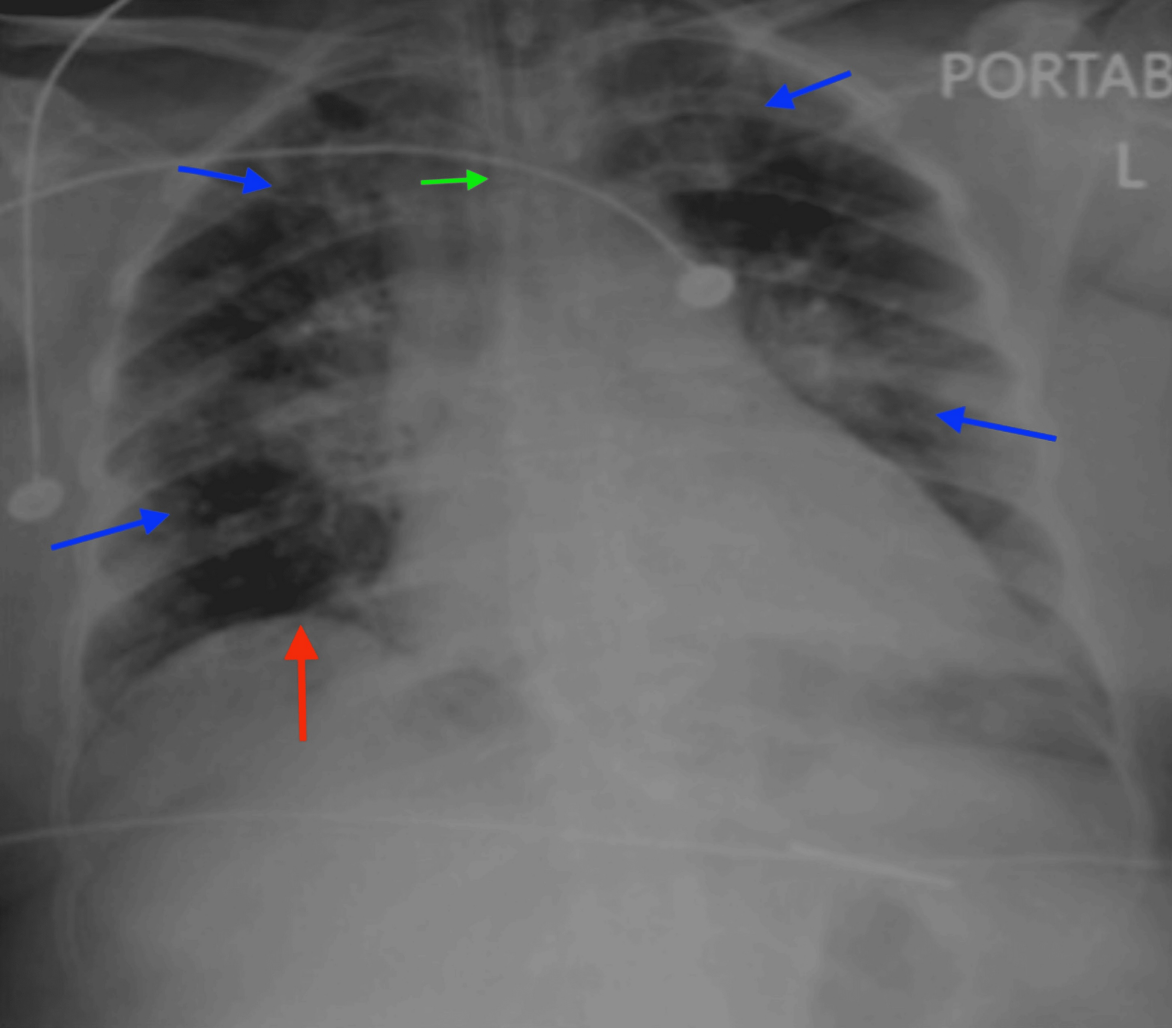
Post-Intubation Chest Radiograph Post-intubation chest radiograph demonstrating appropriate endotracheal tube placement and progression of bilateral pulmonary edema. The red arrow indicates persistent elevation of the right hemidiaphragm. The blue arrows indicate new bilateral interstitial and alveolar opacities consistent with pulmonary edema. The green arrow indicates the endotracheal tube in appropriate position.

Imaging studies demonstrated a normal pre-arthroscopy chest radiograph without acute intrathoracic pathology (Figure 1). A pre-intubation chest radiograph revealed elevation of the right hemidiaphragm, suggestive of ipsilateral phrenic nerve involvement (Figure 2). Following intubation, repeat chest radiography demonstrated new bilateral pulmonary edema with appropriate positioning of the endotracheal tube (Figure 3).

**Figure 4:**
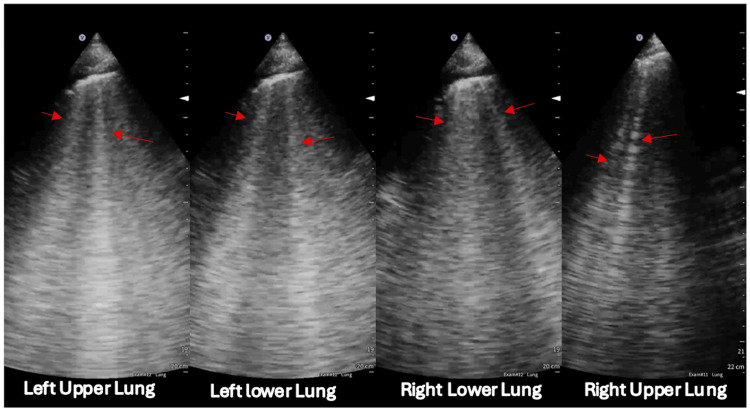
Lung Ultrasound Examination Lung ultrasound images obtained from the left upper, left lower, right lower, and right upper lung zones demonstrate multiple confluent vertical hyperechoic artifacts (B-lines) arising from the pleural line and extending to the bottom of the screen without fading (red arrows). Findings are present bilaterally across multiple lung fields most consistent with acute pulmonary edema.

Point-of-care ultrasound (POCUS) demonstrated diffuse bilateral B-lines consistent with pulmonary edema (Figure 4). There was no evidence of right ventricular strain, pulmonary embolism, or pericardial tamponade.

Hospital course

The patient was managed with mechanical ventilation and supportive care. Pulmonary edema improved with intravenous loop diuretics, achieving a net negative fluid balance of approximately 1.5 L. Transthoracic echocardiogram demonstrated normal cardiac function with no evidence of valvular disease or structural abnormalities (Video 1).

**Video 1:** Transthoracic Echocardiography Transthoracic echocardiography video demonstrating mildly reduced systolic function without significant structural or valvular abnormalities.

**Figure 5:**
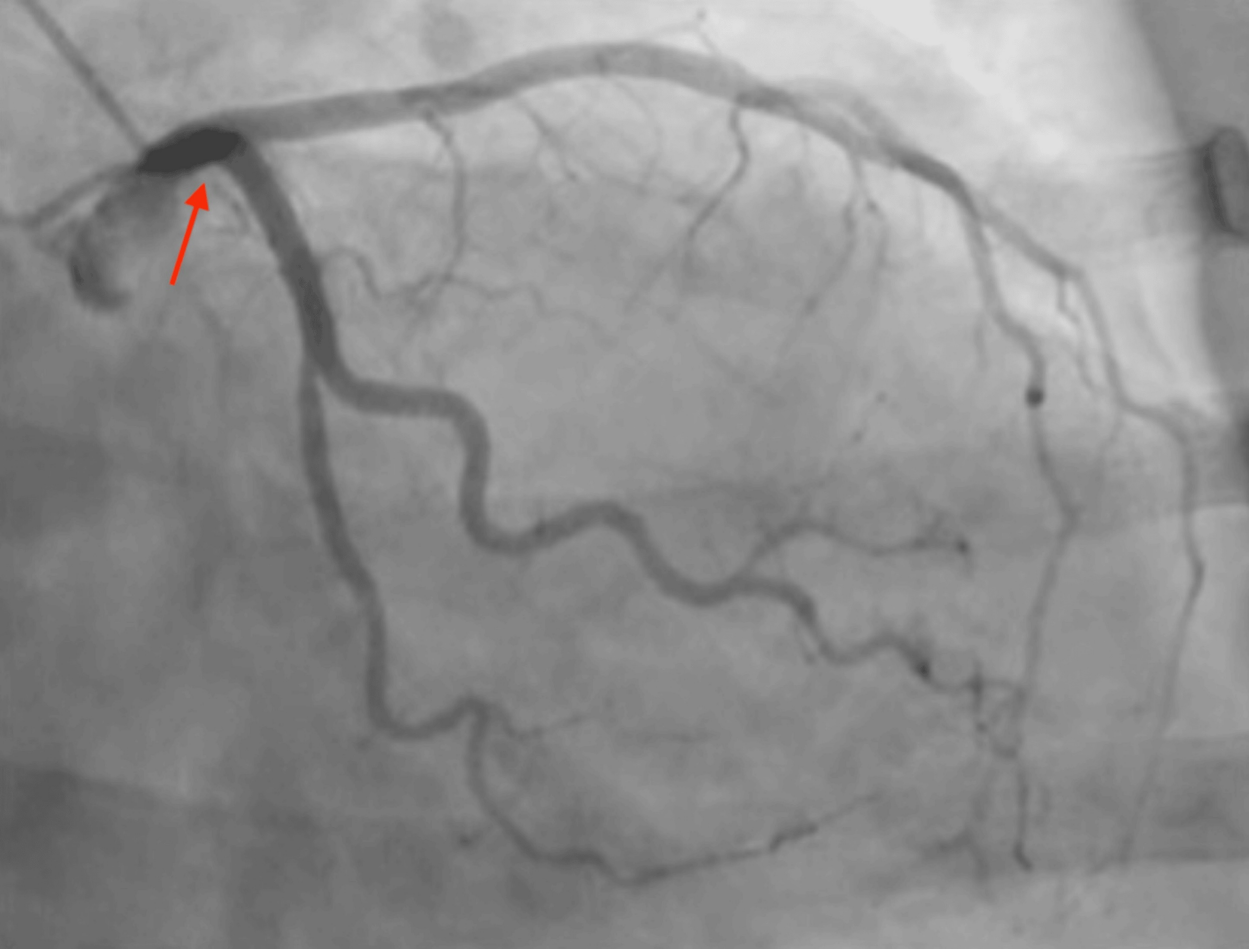
Left Coronary Angiography Left coronary angiography demonstrating nonobstructive coronary artery disease without significant stenosis. The red arrow identifies the left coronary arterial system.

**Figure 6:**
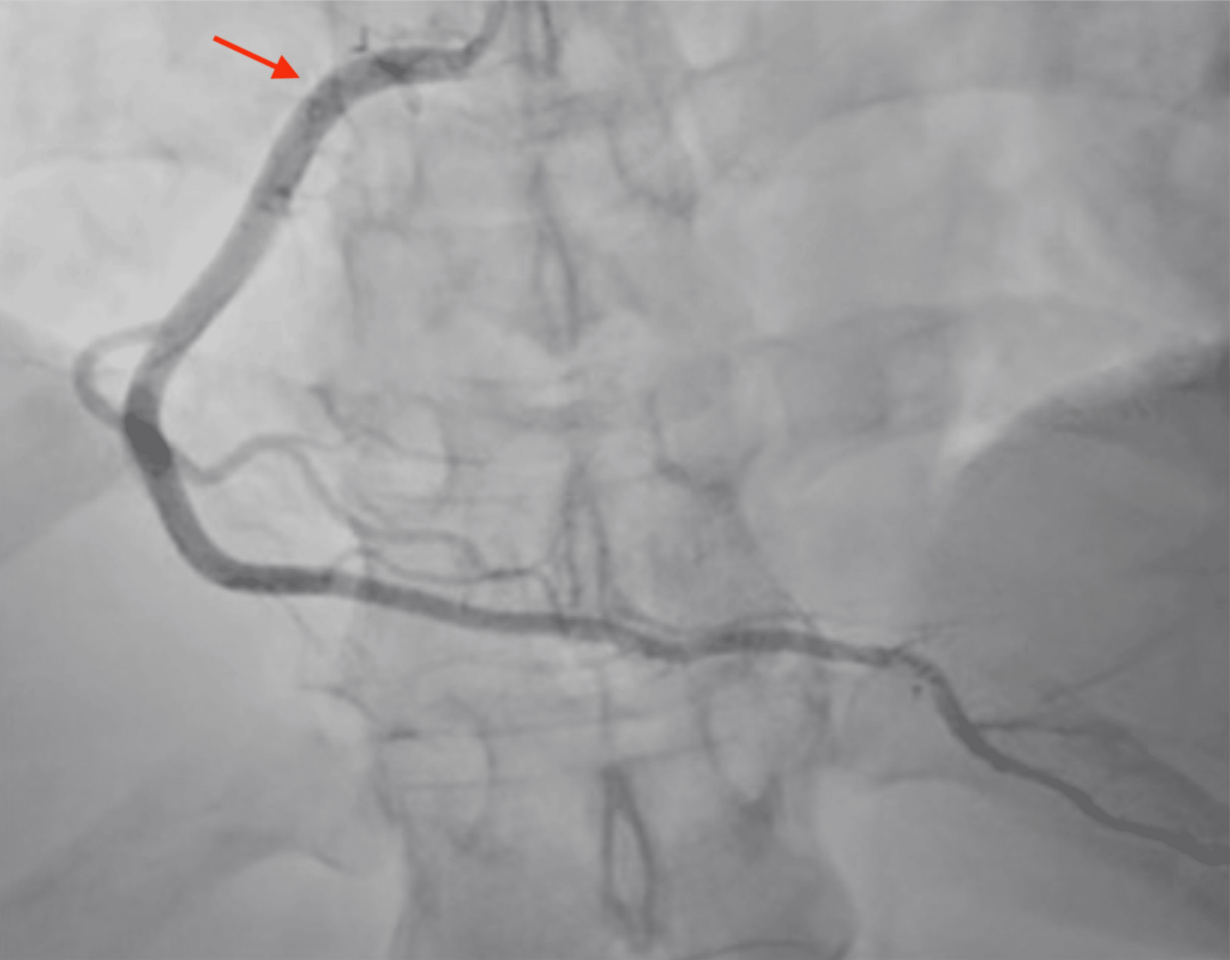
Right Coronary Angiography Right coronary angiography demonstrating nonobstructive coronary artery disease without significant stenosis. The red arrow identifies the right coronary arterial system.

Given concern for acute coronary syndrome in the setting of elevated troponin levels, the cardiac catheterization laboratory was activated, and coronary angiography was performed, revealing non-obstructive coronary artery disease (Figures 5, 6, Video 2).

**Video 2:** Coronary Angiography Coronary angiography video demonstrating no significant coronary artery lesions.

Over the subsequent 24 hours, the patient's ventilatory requirements decreased substantially, and spontaneous breathing trials were initiated. By 48 hours, she successfully tolerated spontaneous breathing trials and was extubated. She was maintained on bilevel positive airway pressure (BiPAP) support for approximately two hours following extubation before being transitioned to room air. Arterial blood gases and repeat laboratory studies remained reassuring, and the patient experienced no recurrence of respiratory symptoms (Tables 3, 4). She was subsequently transferred from the intensive care unit to the general medical ward.

**Table 3:** Serial Arterial Blood Gas Measurements and Respiratory Support During Hospitalization Serial arterial blood gas analyses obtained throughout the hospital course, demonstrating the evolution of gas exchange during acute postoperative respiratory failure, mechanical ventilation, ventilator weaning, and post-extubation recovery. Initial ABG analysis demonstrated hypoxemia despite high-concentration oxygen delivered via a non-rebreather mask. Following endotracheal intubation and mechanical ventilation, oxygenation improved markedly. Subsequent studies showed progressive normalization of gas exchange, successful liberation from mechanical ventilation, and maintenance of adequate oxygenation on room air 24 hours after extubation. ABG: arterial blood gas analysis; PaCO₂: arterial partial pressure of carbon dioxide; PaO₂: arterial partial pressure of oxygen; SaO₂: arterial oxygen saturation; CMV: continuous mandatory ventilation; NIV: noninvasive ventilation; BIPAP: bilevel positive airway pressure; IPAP: inspiratory positive airway pressure; EPAP: expiratory positive airway pressure; PSup: pressure support; PEEP: positive end-expiratory pressure; FiO₂: fraction of inspired oxygen; TV: tidal volume; RR: respiratory rate.

Parameters	ABG #1 (Day #1)	ABG #2 (Day #1)	ABG #3 (Day #2)	ABG #4 (Day #3)	ABG #5 ( Day #3)	ABG #6 (Day #4)	Reference Range
pH	7.36	7.339 - Low	7.461 - High	7.466 - High	7.469 - High	7.461 - High	7.350 – 7.450
PaCO_2_	41.9	46.9 - High	33.1 - Low	34.2 - Low	35.8	33.0 - Low	35.0 – 45.0 mmHg
PaO_2_	88	316.8 - High	170.3 - High	102.6 - High	127.7 - High	86.6	80.0 – 100.0 mmHg
HCO_3_	23.1	22.4	23.2	24.1	25.4	23	22.0 – 26.0 mmol/L
SaO_2_	94.2- Low	99.7	99.6	98.3	99.1	97.1	95.0 – 100.0 %
O_2 _Delivery Method	Non-rebreather mask	Mechanical ventilation	Mechanical ventilation	Mechanical ventilation	NIV	Room Air	
Respiratory support settings	At 15 L/min (approx. 95-100% FiO_2_)	Mode CMV, TV 500, FiO_2_100%, PEEP 5, RR 16	Mode CMV, TV 500, FiO_2_60%, PEEP 5, RR 16	Mode Spontaneous, Psup10, FiO_2_40%, PEEP 5	Mode BiPAP, IPAP 12, EPAP 6, FiO_2_40%, RR 14	FiO_2_ 21%	
Comments	15 min postoperative	30 min after initiation of mechanical ventilation	24 h after initiation of mechanical ventilation	48 h after initiation of mechanical ventilation	2 h post- extubation	24 h post- extubation	

**Table 4:** Interpretation of Serial Arterial Blood Gas Analyses During Hospitalization Clinical interpretation of serial arterial blood gas analyses demonstrating the progression of acute postoperative respiratory failure, response to mechanical ventilation, ventilator weaning, and recovery following extubation. ABG: arterial blood gas analysis.

ABGs	Interpretation
ABG #1	Mild hypoxemia despite high-concentration oxygen therapy, consistent with acute hypoxemic respiratory failure secondary to upper airway obstruction.
ABG #2	Marked improvement in oxygenation following emergent intubation and initiation of mechanical ventilation, with mild respiratory acidosis reflecting peri-intubation hypoventilation.
ABG #3	Significant recovery of gas exchange with resolution of respiratory acidosis and development of mild respiratory alkalosis, with persistent hyperoxemia on reduced FiO_2_.
ABG #4	Stable oxygenation and ventilation during spontaneous breathing trial with mild respiratory alkalosis, supporting readiness for extubation.
ABG #5	Adequate oxygenation maintained following extubation while receiving noninvasive ventilatory support, with persistent mild respiratory alkalosis.
ABG #6	Near-normal gas exchange on room air with mild residual respiratory alkalosis, consistent with recovery from acute respiratory failure.

At 72 hours, the patient remained clinically stable without recurrence of respiratory distress or hypoxemia and was discharged home in stable condition.

## Discussion

The rarity of this presentation lies not only in the occurrence of bilateral recurrent laryngeal nerve dysfunction following interscalene brachial plexus block, but also in the cascade of respiratory and cardiovascular complications that subsequently developed. The patient's clinical course is best explained by a sequence of interconnected pathophysiologic events initiated by unintended involvement of adjacent neural structures. Understanding the underlying anatomy and physiologic responses to acute upper airway obstruction provides a framework for explaining the progression from bilateral vocal cord paralysis to negative-pressure pulmonary edema and secondary type 2 myocardial infarction.

Vocal cord dysfunction and differential diagnosis of airway obstruction 

The recurrent laryngeal nerves provide motor innervation to all intrinsic laryngeal muscles except the cricothyroid muscle and are essential for vocal cord abduction and maintenance of airway patency [9]. Consequently, bilateral recurrent laryngeal nerve dysfunction can impair vocal cord mobility and result in fixation of the vocal cords in a paramedian position. This configuration prevents adequate abduction during inspiration and creates a fixed upper airway obstruction [5,6].This mechanism explains the marked resistance encountered during bag-valve-mask ventilation, severe hypoxemia despite supplemental oxygen administration, and the rapid improvement in oxygenation and ventilation following endotracheal intubation. In contrast to unilateral vocal cord paralysis, which is often compensated and may present primarily with dysphonia, bilateral involvement can result in critical airway narrowing and rapid respiratory decompensation.

Laryngospasm was considered in the differential diagnosis because it may also present with acute upper airway obstruction and severe hypoxemia. However, laryngospasm is typically a transient phenomenon caused by reflex glottic closure that resolves with positive-pressure ventilation, deepening anesthesia, or administration of neuromuscular blockade. In this case, marked resistance to ventilation persisted, and direct laryngoscopy demonstrated vocal cords fixed in an adducted (paramedian) position. Notably, the vocal cord position remained unchanged following administration of both sedative and paralytic agents, findings that strongly argue against laryngospasm and support bilateral vocal cord paralysis as the underlying etiology.

Proposed mechanism of nerve involvement

The mechanism of this complication is best explained as a sequential process involving anatomical proximity and procedural factors.

The interscalene block targets the C5-C6 nerve roots within the interscalene groove, which lies in proximity to the phrenic nerve anteriorly and the recurrent laryngeal nerve medially. This anatomical relationship predisposes these structures to unintended anesthetic spread [1,5].

Following injection, the use of large volumes of pressurized irrigation fluid during shoulder arthroscopy may have resulted in soft tissue extravasation, increasing local tissue pressure, and disrupting normal fascial planes. This may have facilitated medial tracking of the anesthetic solution beyond its intended distribution.

As a result, the anesthetic agent first affected the ipsilateral phrenic nerve, causing hemidiaphragmatic paresis, and subsequently extended medially to involve the ipsilateral recurrent laryngeal nerve [1,5,8]. Continued spread across the midline may have resulted in contralateral recurrent laryngeal nerve involvement, producing bilateral vocal cord paralysis.

This sequential progression provides a plausible explanation for the rarity and severity of this presentation.

Mechanism of pulmonary edema

The pulmonary edema was attributed to negative-pressure pulmonary edema secondary to acute upper airway obstruction. Vigorous inspiratory efforts against an occluded airway produced markedly negative intrathoracic pressures, resulting in increased venous return, elevated pulmonary capillary hydrostatic pressures, and subsequent transudation of fluid into the alveoli. Concurrent hypoxemia triggered sympathetic activation, which further increased pulmonary vascular pressures and capillary permeability, amplifying the development of pulmonary edema [5,6].

Mechanism of type 2 myocardial infarction

The troponin elevation was most consistent with a type 2 myocardial infarction resulting from profound hypoxemia, increased respiratory effort, and catecholamine-mediated sympathetic activation, leading to myocardial oxygen supply-demand mismatch [14]. The lack of ischemic electrocardiographic findings and the demonstration of nonobstructive coronary artery disease on coronary angiography favored demand ischemia over an acute atherothrombotic coronary syndrome.

Patient-specific risk factors

Patient-specific factors, including obesity and crowded oropharyngeal anatomy, may have predisposed her to a reduced baseline airway caliber, increased airway collapsibility, and heightened vulnerability to upper airway obstruction. These anatomic characteristics may have exacerbated the degree of airway compromise caused by bilateral vocal cord paralysis, contributing to the severity of the clinical presentation.

## Conclusions

This case highlights a rare but potentially life-threatening complication of interscalene brachial plexus block in which unintended anesthetic spread resulted in bilateral recurrent laryngeal nerve dysfunction, acute upper airway obstruction, negative-pressure pulmonary edema, and secondary type 2 myocardial infarction. Although phrenic nerve paresis and transient vocal cord dysfunction are recognized complications of interscalene block, progression to bilateral vocal cord paralysis with severe respiratory compromise is exceedingly uncommon. Clinical recognition may be challenging because the initial presentation can mimic more common postoperative respiratory events, including laryngospasm, opioid-induced hypoventilation, and residual neuromuscular blockade. In patients who develop unexpected resistance to ventilation, persistent hypoxemia, or signs of upper airway obstruction following interscalene brachial plexus block, bilateral vocal cord dysfunction should be considered in the differential diagnosis. Early airway evaluation and prompt airway stabilization are essential to prevent progression to severe respiratory and cardiovascular complications, improving patient outcomes.
